# An Image-Voice Dietary Assessment System for Estimating Individual Nutrient Intakes in Cambodian Women and Children: Relative Validity, Reliability, and Acceptability Study

**DOI:** 10.2196/65939

**Published:** 2025-09-17

**Authors:** Megan E Rollo, Janelle L Windus, Samantha J Stewart, Connor T Dodd, Marc T P Adam, Kerith Duncanson, Tracy L Burrows, Kim Colyvas, Clare E Collins

**Affiliations:** 1 School of Health Sciences College of Health, Medicine and Wellbeing University of Newcastle Callaghan Australia; 2 School of Population Health Faculty of Health Sciences Curtin University Bentley Australia; 3 Food and Nutrition Research Program Hunter Medical Research Institute New Lambton Australia; 4 School of Information and Physical Sciences College of Engineering, Science and Environment University of Newcastle Callaghan Australia; 5 School of Medicine and Public Health College of Health, Medicine and Wellbeing University of Newcastle Callaghan Australia

**Keywords:** acceptability, dietary intake assessment, image-based food records, relative validity, reliability

## Abstract

**Background:**

Individual-level dietary intake data are fundamental for developing nutrition policy and programs. In low- and lower-middle-income countries, proxy measures of individual intake (household consumption and expenditure surveys and food balance sheets) are often used, with limited implementation of new technology-assisted applications.

**Objective:**

We aimed to determine the relative validity, test-retest reliability, and acceptability of the Voice-Image Solution for Individual Dietary Assessment (VISIDA) system in a sample of Cambodian women and their children aged ≤5 years.

**Methods:**

Mothers and one of their children were recruited from 3 locations (rural, semirural, and urban) in Siem Reap province, Cambodia. Dietary intake data were collected for each participant using 2 methods across 3 recording periods over approximately 4 weeks. In week 1, intake was recorded using VISIDA for 3 nonconsecutive days, followed by 3 interviewer-administered, multiple-pass 24-hour recalls collected in weeks 2 to 3. In week 4, VISIDA was used again to collect a 3-day food record. After the third intake recording period, the mothers completed a feedback survey. Differences in estimated nutrient intakes for the 3 recording periods for mothers and children were examined using a linear mixed model approach.

**Results:**

The analysis included 210 participants (n=119, 56.7% mothers and n=91, 43.3% children). Estimated mean nutrient intakes reported in both VISIDA recording periods were mostly lower compared to intakes reported using the 24-hour recalls. Compared to the 24-hour recalls, statistically significant differences were found for the VISIDA recording periods for 80% (16/20) of nutrients for mothers and 32% (6/19) of nutrients for children. Nutrient intakes estimated from both VISIDA recording periods showed no statistically significant differences for mothers and children. For mothers, the differences of model weighted marginal means in energy intakes (kcal) were −296 (95% CI −410 to −181; VISIDA period 1 minus 24-h recall), −274 (95% CI −390 to −158; VISIDA period 2 minus 24-h recall), and −22 (95% CI −131 to 87; VISIDA period 1 minus VISIDA period 2). For children, the differences in model weighted marginal means in energy intakes (kcal) were −158 (95% CI −227 to −89; VISIDA period 1 minus 24-h recall), −127 (95% CI −198 to −57; VISIDA period 2 minus 24-h recall), and −31 (95% CI −98 to 37; VISIDA period 1 minus VISIDA period 2). Most mothers reported that the VISIDA smartphone app was “easy to use” (68/108, 63%) or “very easy to use” (23/108, 21.3%) for collecting dietary intake data.

**Conclusions:**

The VISIDA system produced lower estimates of nutrient intakes when compared to the 24-hour recalls in a sample of mothers and children in Siem Reap province, Cambodia. However, the estimated nutrient intakes for the 2 VISIDA recording periods were similar. The participating mothers reported high acceptability for using the VISIDA smartphone app to collect intake data.

## Introduction

### Background

Globally, suboptimal diet is the leading risk factor associated with mortality, with low intakes of whole grains and fruits and high intake of sodium associated with 50% of deaths across 195 countries between 1990 and 2017 [1]. Low- and lower-middle-income countries (LLMICs) experience the unique challenge of a double burden of malnutrition where undernutrition and overweight, obesity, and diet-related noncommunicable diseases are increasing concurrently [2]. This double burden of malnutrition is driven by a nutrition transition in LLMICs evident by changes in the food supply, such as increased availability of ultraprocessed foods, along with decreased physical activity and increased sedentary behaviors [3].

To inform program and policy developments addressing the double burden of malnutrition in LLMICs, high-quality data on individual-level food and nutrient intakes are essential [4]. In these settings, proxy estimates of individual intake, such as national food balance sheets and household consumption and expenditure surveys, are often used with data sources to inform nutrition support decisions [4,5]. However, these approaches are not considered accurate for estimates of intake at an individual level [6], and do not allow for data to be viewed by sex or age [4,5]. Since 2000, there has been a notable increase in the number of dietary surveys conducted in LLMICs, with most of these surveys completed using 24-hour recalls and data capture with pen and paper [7]. Despite this, barriers to collecting individual-level dietary intakes in LLMICs, such as cost and time, infrastructure, and capacity, continue to exist [4].

The development of web-based 24-hour recalls and smartphone food record apps has streamlined the collection of individual-level food and nutrient intake data over the past 2 decades, with a focus of implementation in high income countries [8]. Bell et al [9] summarized the challenges regarding the implementation of technology-assisted dietary assessment methods in LLMICs. Common barriers included low literacy levels, limited or unreliable network connectivity, a lack of infrastructure for data management, and few formally trained nutritionists [9]. In their review of the suitability of existing technologies to support individual-level dietary assessment in LLMICs, Bell et al [9] found that most of the camera-enabled technologies that captured dietary intake in the form of images showed potential for use in this context as these approaches did not rely on participant literacy, could be used on devices with an appropriate battery life and function without a network connection, and captured sufficient data to quantify macronutrient and micronutrient intakes [9].

In the past 15 years, there has been a rapid rise in the use of intake data in the form of images, accelerated by the increased ubiquity of mobile and smartphones with an embedded digital camera. As such, 2 distinct dietary assessment methods using images have emerged: image-based and image-assisted. Image-based dietary assessment methods aim to capture all eating occasions using images as the primary record of dietary intake and follow food record methodology, with intake data collected prospectively [10]. In contrast, image-assisted methods use the images to assist traditional dietary assessment methods, primarily 24-hour recalls, to aid as a memory prompt and/or to assist in the estimation of portion size of food and drinks consumed [10]. Furthermore, images may be collected actively by the participants themselves or passively through a fixed, mounted camera or a camera worn on the body [10]. Similar to other technology-assisted dietary assessment methods, the implementation of imaging approaches has been primarily focused in high income countries [11,12] with only a few studies having used this methodology in LLMICs [13-15].

A spoken food record is another technology-assisted method, which, to date, has been given limited attention compared to other dietary assessment methods. In this approach, voice recordings containing descriptions of foods consumed or intended for consumption are collected. Advances in and accessibility to natural language processing have led to a rise in the automated processing of speech intake data in recent years [16]. However, despite the potential suitability of spoken food records among users with low literacy, there has been no use of this approach in LLMICs [16].

Dietary assessment research in Cambodia, currently classified as a lower-middle income country, has primarily focused on the assessment of the nutrient adequacy of diets in relation to micronutrient deficiencies [17]. Furthermore, the synthesis of research centered on the application of dietary assessment methods in Cambodia revealed a preference for interviewer-administered 24-hour recall methods, with minimal use of technology to aid in data collection [18].

### Objective

We aimed to describe a new dietary assessment system, the Voice-Image Solution for Individual Dietary Assessment (VISIDA), and to evaluate its relative validity in comparison to 24-hour recalls, as well as its test-retest reliability and acceptability, among Cambodian women and children aged ≤5 years.

## Methods

### Study Design

This study was a free-living, observational design, with data collected between August 2019 and March 2020 in Siem Reap province, Cambodia. Specifically, participants were recruited from three communities within Siem Reap province: (1) Svay Svar commune, Varin (rural); (2) Sragnea commune (semirural), and (3) Siem Reap city (urban). A local nongovernmental organization (NGO), This Life Cambodia, supported logistics and in-country approvals, in addition to completing the data collection and parts of the data processing, following comprehensive training under the direction of the University of Newcastle research team.

### Ethical Considerations

This study was approved by the National Ethics Committee for Health Research in Cambodia (reference number 151 NECHR), along with approval at the province level from the Ministry of Health, Provincial Health Department of Siem Reap, Cambodia. This study was also approved by the University of Newcastle (H-2018-0515) and Curtin University (HRE2022-0366) Human Research Ethics Committees. Informed consent was provided by all participants. The NGO research team first approached the relevant commune community leaders in the 3 sites. The research objectives and methods were described to the community leaders to request their support. The leaders were asked to consider which households may be suitable and interested in participating. Local households were then invited to a group meeting to discuss the research with the mothers from these households. During this meeting, information about the study was presented as either a written document or read aloud to interested individuals. The adult female member of each household who wished to participate provided verbal consent for participation for herself and her child, with this consent recorded via an audio file. Participants were asked to invite eligible extended family and friends to participate, with information on the study then provided to these individuals as previously described. All information about the study, including consent, was provided in the native language of Cambodia, Khmer. Participating households received US $5 per household for each day that they participated in the study. Collected participant data were deidentified.

### Participants

We aimed to recruit 2 participants from each of 150 households (mother and child), with 50 (33.3%) households each recruited from the rural, semirural, and urban communities in Siem Reap province. A sample size of 150 households was calculated based on equivalent relative validity studies [11], in conjunction with pragmatic considerations for study implementation. To be eligible to participate, each household was required to include a female adult, aged ≥18 years, who was a mother with one child aged ≤5 years. Breastfeeding mothers and their breastfed infants were eligible to participate. Pregnant women were ineligible to participate.

### Data Collection

#### Overview

Each participating mother and child completed data collection as a household over approximately a 4-week period. Demographic and household inventory data were captured at the start of the data collection period and before the collection of dietary intake data. Food and beverage intake data were recorded using two methods over three recording periods in the following order: (1) the VISIDA image-voice food record (IVFR) smartphone app over 3 days (week 1), (2) three 24-hour recalls (weeks 2-3), and (3) the VISIDA IVFR smartphone app again for 3 days (week 4). All dietary intake recording days in each of the 3 recording periods were nonconsecutive and included one weekend day to account for any variation in intake on weekend days compared to weekdays. At the conclusion of the intake data collection, the participating mothers completed a feedback survey. Multimedia Appendix 1 provides an overview of the data collection sequence for participants, and a detailed description of the methods used for data collection and processing of intake data are provided in subsequent sections.

#### Demographic and Household Inventory Data

Demographic information, including age, household composition, education level, occupation, smartphone use for the mother, and age and gender for the participating child, were collected before the collection of intake data. Data were collected via an interviewer-administered questionnaire completed with the participating mother by the research assistants using a paper-based form in the field and later entered into an online form. In addition, research assistants completed a household inventory for each participating household where images, dimensions and capacity measures of usual serving and eating vessels, and utensils used in each participating household and by the participating individuals were recorded. The images of key household eating vessels were captured by the research assistants following a standardized process using the VISIDA smartphone app, as they were to be used to assist with the processing of the collected dietary intake data.

#### Dietary Intake Data Collection, Processing, and Analysis

##### Overview

Two dietary assessment methods were used for the collection of dietary intake data: the VISIDA system and 24-hour recalls. In this study, the VISIDA system was considered the “test method” with the relative validity of this new method to be evaluated through comparison to intake estimated from the “reference method” of 24-hour recalls. Key issues relating to the design of the relative validity component of the study were considered [19-21]. The 24-hour recall method was selected as the reference method as, although it is a self-report method, it is widely acknowledged to have the least amount of bias compared to food frequency questionnaires [22,23] and has been used in relative validity studies involving dietary assessment methods [21]. To maintain independence between the administration of the methods to establish relative validity, intake data were not collected concurrently [20] with the VISIDA method, as the test method was administered first before the 24-hour recalls. In addition, the subsequent recording periods were scheduled to allow all intake data collected within an approximate 4-week period to minimize any potential effects relating to seasonality within one household. The selection of 3 nonconsecutive recording days for each recording period was made for pragmatic reasons (eg, to minimize participant burden) and follows a similar approach to another relative validity study [24] involving image-based food record conducted by the research team. The test-retest reliability of the VISIDA was determined through comparison of estimates of nutrient intake collected in weeks 1 and 4 to ensure independence between repeat administrations of the method [19].

#### The VISIDA System

##### Overview

**Figure 1:**
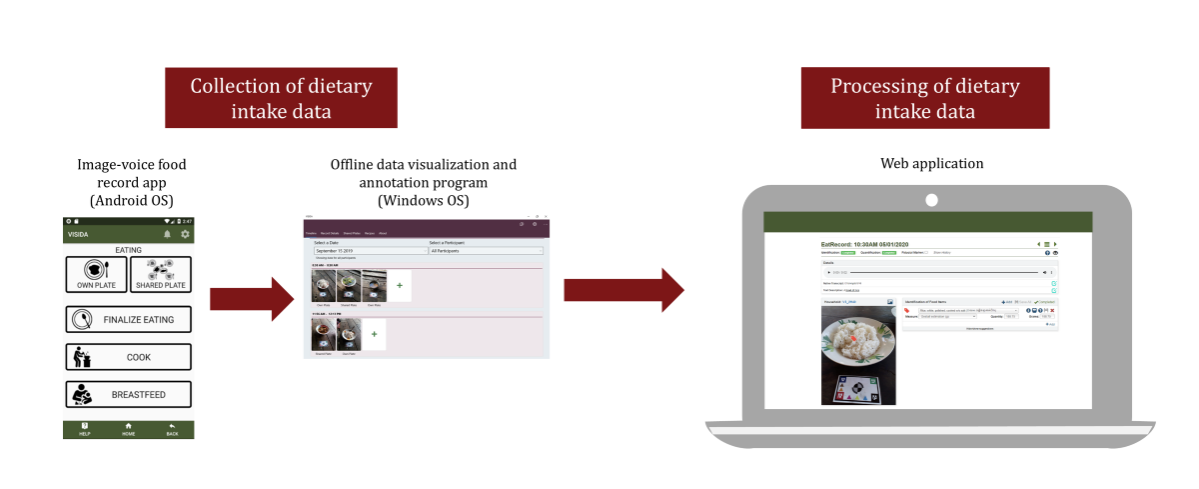
The Voice-Image Solution for Individual Dietary Assessment (VISIDA) system components.

The VISIDA system is a multicomponent platform that allows for the collection, processing, analysis, and interpretation of individual-level dietary intake data via images and voice recordings [25]. The system has been developed for use in LLMICs and accounts for the unique challenges associated with using technology-assisted methods in these settings. Another unique aspect of the VISIDA system is that it can capture food consumed from a shared plate or bowl where the contents of the food served are consumed by 2 or more individuals. Shared plate eating is common in LLMICs; however, it is an area of dietary intake assessment with limited research [26,27]. The VISIDA system development was informed by previous image-based dietary assessment work of the research team [24,28-30], in addition to elements of co-design [31]. The development of this system occurred through an iterative process with formative work completed internally. The version of the VISIDA system used in this study comprised an Android smartphone app for the collection of intake data via an IVFR, an offline program for viewing and annotating collected intake data, and a web application for the semiautomated processing and analysis of the collected intake data (Figure 1 shows the English language version of IVFR app; see a previous study [32] for an overview of the design of the web application component).

##### Collection of Intake Data Using the VISIDA System

In this study, the intake data were collected at the individual level using the VISIDA IVFR app, with the participating mother responsible for collecting intake and recipe data for herself and her participating child. The mother was trained to use the IVFR app and was provided with a smartphone (Sony Xperia L1) installed with the Khmer language version of the IVFR app for each VISIDA data collection period. The IVFR app also contained audio-visual help screens and short videos in Khmer to support users in collecting intake data during the recording days. A printed visual summary of the recording steps was provided to each mother. In addition, each mother was provided with a small waist bag that they could use to carry the study phone throughout the day if they desired, along with spare fiducial markers (a card of known dimensions). The day following the initial training, each mother completed a test recording day where they were asked to use the app to record all food and drinks consumed over the day for themselves and their participating child. The research assistant provided feedback to the participant during and following this test recording day. Data from this day were not used in the analysis.

For eating occasions, images and voice recordings documenting dietary intake were collected before eating. The IVFR app was used to capture data for shared servings (designated as “shared plate” in the smartphone app) and discrete servings (“own plate”) at eating occasions for each participant. When capturing an image of food items, mothers were instructed to assemble the food items so that all items were clearly visible and to place a fiducial marker next to the food items. On-screen guidance within the IVFR app assisted the user in positioning the marker in a consistent position (approximately 45° angle) when capturing an image. After capturing an image, the mother collected a voice recording briefly describing the contents of the image. The eating occasions for each participant were finalized by the mother via the IVFR app by indicating if each food item was eaten completely (ie, no leftovers), was not consumed, or only partially consumed (ie, leftovers were present). For any leftover food, the mother captured another image and voice recording to document the remaining food. For shared servings, the mother was asked to indicate which study participants (herself or her child) ate from the serving, along with the total number of adults (both male and female) and children who ate from the shared serving. For foods prepared in the home, recipe information was collected in the form of images and voice recordings for the ingredients and the final prepared dish. At the end of a recording day, the mother was prompted by the app to review the intake data for the day for herself and her participating child. If any food items were eaten but not recorded in the app, they were asked to make a voice recording with the details (description and estimated amount) of the forgotten food items.

Following each of the recording days, research assistants visited the participants in their homes and viewed the previous day’s intake data collected using the IVFR app. This data quality check process allowed for the collection of any additional information to supplement the IVFR app data to maximize completeness. Any changes to the intake data were documented in the field by the research team. At the completion of the data collection period, data from the quality check process were transferred into the VISIDA system’s data viewer and annotator program (Figure 1) along with the IVFR app data to produce a consolidated version of the dietary intake data for participants in each household. The intake data were then exported for processing and analysis within the VISIDA web application.

##### Processing and Analysis of Intake Data Within the VISIDA System

The dietary intake data were uploaded into a web application (Figure 1) for processing to produce estimates of nutrient intake using a food composition database (FCD). Processing of the imported image and voice recording intake data within the web application was semiautomated, with several system features to support an analyst (eg, nutritionist, research assistant, or field worker) trained in using the system to identify and quantify the intake data. For example, the web application automatically transcribed and translated the voice recordings using the Google Translate API [33], and then automatically matched to items in the selected FCD and offered suggestions for the analyst to review, or alternatively, to manually search the FCD for a more suitable match.

In-country Khmer-speaking research assistants from the partner NGO were trained to assist with the identification stage of the processing of the intake data, with verification by a Khmer-speaking dietitian (JLW). Ingredients in recipes and food items recorded in the eating occasions were matched to the items within a Cambodian FCD. In addition, the research assistants entered quantities that were present in the image (eg, for packaged food) or voice recording (eg, weight of ingredients as purchased by weight at the local market). For the remaining quantities, members of the University of Newcastle research team (SJS, JLW, or MER), trained as analysts, estimated the portion sizes of the food items contained in the images.

The portion size estimations were assisted through the use of aids within the VISIDA web application, which included (1) a reference image database, consisting of images of >90 food items common in Cambodia or recognized as difficult to quantify (eg, cooked rice)food items in different portions of a known weight, (2) measures for >300 food items within the system’s Cambodian FCD, and (3) a virtual ruler, calibrated via the fiducial marker, that could be used to estimate dimensions of food items and serving vessels. In addition, the size and capacity of common serving vessels documented in the relevant household’s inventory were also available to assist with estimating portion size. Where quantities were estimated from the image, 2 analysts independently estimated the portion of the relevant food item, with estimates blinded. For food items with estimates of portion size that differed by more than 25% between analysts, a third analyst reviewed the record and made a final decision on the quantity. When a quantity could not be estimated from the data provided, a median portion size of the same or similar food consumed by the individual participant or shared serving was used, where available. If this data were not available in the collected intake data, a median portion size was calculated for the portion sizes for the same or similar food items from the same participant type (ie, mother or child) and used.

Once processed by analysts, recipes collected for a household were presented as identification options to the analyst within the system for all eating occasions in the same household. For recipes, nutrient retention factors at the ingredient level were automatically applied, and a nutrient profile (per 100 g) was generated for the final prepared recipe. For servings that were shared, the total amount of the food item eaten was proportioned evenly among the total number of people eating the food item and then assigned to the participant if they were eating the dish. For example, an adult female participant who ate from a shared plate along with an adult female nonparticipant and a male nonparticipant would be assigned one-third of the total amount consumed.

#### Interviewer-Administered 24-Hour Recall

In the second and third weeks, three 24-hour recalls were conducted in person by the trained in-country research assistants at each participating adult female’s home. The three recall days were nonconsecutive and included one weekend day. The mother reported her intake, followed by the intake of her participating child. An interviewer-administered, multiple-pass 24-hour recall was adapted from a previous study [24] and used the following passes: (1) a quick list of all items consumed in the previous 24-hour period followed by a checklist for forgotten foods; (2) detail on foods recalled (ie, amounts, type, cooking or preparation methods, and leftovers); and (3) review of recalled food items.

The quantities of foods consumed were estimated using one of three approaches: (1) a reference food image database comprising 23 common foods, with 4 images for each food representing different portion sizes along a continuum; (2) common household serving utensils for Cambodia, such as a rice serving spoon, *somlar* (or “eating spoon”), or coffee spoon (or teaspoon); and (3) the size of the package for packaged foods. During the recall interview, the research assistants read from a script to ensure a standardized process was followed. All recalls were completed in Khmer. In the field, data were collected with pen and paper, translated to English, and then transferred into a purpose-built Microsoft Access database. Data were then exported and loaded into the VISIDA web application. The data contained in the recalls were then coded within the web application, with the descriptions of the food items matched to an appropriate item in the Cambodian FCD and the quantities entered.

#### Cambodian FCD

As no comprehensive Cambodia-specific FCD was available at the commencement of the project, the research team undertook a systematic process to develop and compile an FCD that represented the usual composition of foods in the forms commonly consumed in Cambodia. The process for compiling the new Cambodian FCD followed the Food and Agriculture Organization and the International Network of Food Data Systems guidelines and associated training materials [34]. Following evaluation of relevant published FCDs, primary food composition data from the SMILING (Sustainable Management of Iodine and other Micronutrients through Integrated Local and Global Efforts) Cambodian [35] and the Association of Southeast Asian Nations [36] FCDs were included. Food composition data borrowed from secondary data sources included the national FCDs of Australia [37,38], the United States [39], and Japan [40] to complete the list of representative foods and supplement missing nutrient values. Following this, a list of foods, categorized into food groups, was prepared by a dietitian and member of the research team (JLW) who had been residing in Cambodia. On the basis of the information obtained during the evaluation, a final list of representative foods and dishes was prepared. Accordingly, local recipes representative of typical Khmer mixed dishes were sourced. Compilation of the nutrient profile data for the food items in the list was managed using the International Network of Food Data Systems Compilation Tool (version 1.2.1) [41]. The final FCD contained nutrient values for 1099 foods (raw and cooked foods) and beverages, including 230 recipes for mixed dishes.

#### Acceptability Data

Following the completion of the third and final dietary intake collection period, the participating mother was asked to complete a brief interviewer-administered questionnaire, with the research assistant collecting data in a similar manner to the demographic questionnaire. Data were collected on the participating mothers’ experiences with using the IVFR app to capture intake data. The participants were asked to report their level of agreement on a 5-point Likert scale (1=strongly disagree to 5=strongly agree) on 20 statements relating to the ease of using the IVFR app to collect dietary intake data for themselves and their participating child, as well as using specific features of the app.

#### Statistical Analysis

To be included in the dietary intake analysis, participants needed to have data for at least 2 of the 3 recording periods, with 2 or 3 recording days of food and beverage intake data collected for each recording period. Due to the aim of this study, only participating children with food and beverage intake data were included in the analysis. Energy, macronutrient, and micronutrient data were included in the analysis, with 20 and 19 nutrients analyzed for mothers and children, respectively. Bland-Altman plots [42], were initially used to visualize any bias and limits of agreement between the methods for mothers and children. As no trends were observed between the difference scores against the mean of the observation pairs as judged by the Spearman nonparametric correlation coefficients, that is, the bias (difference between pairs of observation) was constant and did not vary systematically way over the range of the data, analysis using a linear mixed model approach was appropriate. The linear mixed model was used to examine the differences between the means for the 3 recording periods, with the analysis completed separately for the mothers and children. A fixed effect based on the grouping variable, recording period (levels VISIDA period 1, 24-hour recall, and VISIDA period 2), was used in all models to assess the significance of differences between the dietary intake assessment methods. For accurate estimates of uncertainty in the estimated marginal means for each period, the hierarchical (multilevel) nature of the study design was taken into account by adding 2 random intercepts to the model, one for each study participant and one for each recording period nested within each participant. Residual diagnostics were used to check the assumptions of normality and constant variance. Nonconstant variance was indicated for all components, and regressions were carried out to determine functions for the variability of the residuals by fitting a linear regression to the absolute value of the residuals against the predicted values. The fitted lines estimated the SD of the residuals as a function of predicted value [43]. The SD functions for each intake measure were used to generate weights (1/SD^2^) that were used in fitting a second linear mixed model. Standardized residuals plots (residual/SD) from these models were examined as part of determining the suitability of the SD function used for the weights. A second iteration of this process was carried out with the residuals from the weighted linear mixed model fit being used to determine 2 additional SD functions: one using a linear regression function between the absolute value of the residuals from the weighted model and predicted value, and the other using a cubic regression function. These 2 additional SD functions were used to determine weights for fitting 2 additional weighted linear mixed models. The final model used for reporting results was the one chosen from 4 models fitted to have the most suitable weighting function. The effect sizes reported were for all 3 pairwise differences between the weighted marginal means from the final model, with 95% CIs for the differences. Statistical significance was set at *P*=.05 level. The linear mixed models were fit with SPSS software (version 28.0; IBM) using the MIXED procedure. Descriptive statistics were used to report the findings of the acceptability survey evaluating the IVFR app. Reporting of this study aligns with the STROBE-nut (Strengthening the Reporting of Observational Studies in Epidemiology-Nutritional Epidemiology) checklist [44] (Multimedia Appendix 2).

## Results

### Participant Characteristics

Of the 148 households (comprising a mother and child) starting the study, 41 (27.7%) withdrew at various points throughout the data collection period. Reasons for withdrawal provided for 88% (36/41) of the households included being withdraw by the fieldwork team due to the COVID-19 pandemic (19/36, 53%) or noncompliance with protocol or logistics (7/36, 19%), while reasons for participants actively withdrawing themselves were due to family or personal (5/36, 14%) obligations, new work commitments (3/36, 8%), relocation 1/36, 3%), or illness (1/36, 3%).

Of the 242 participants (children and mothers combined) who had dietary intake data collected during the study, 210 (86.8%) met the analysis inclusion criteria and were included in the final dietary intake analysis. Most households included in the analysis were from the rural site 47/119, 39.5%), followed by the urban (44/119, 37%) location, with a smaller number of participants from the semirural (28/119, 23.5%) location resulting from data collection ceasing early due to the COVID-19 pandemic. Of the mothers included in the analysis (119/210, 56.7%), demographic data was collected for 111 mothers. The age of mothers ranged between 18 and 49 years (mean 28.8, SD 6.0 years), with 50.5% (56/111) completing primary school only. Ownership of mobile phones was reported by 73% (81/111) of the mothers, with the majority (59/81, 73%) of them reporting owning an Android phone. Most mothers (71/81, 88%) reported that their knowledge of mobile phone use was moderate to high, and that they regularly communicated through voice calls (59/81, 73%) and chat-based apps (46/81, 57%). The most common mobile phone apps used by the mothers were Facebook (64/81, 79%) and YouTube (60/81, 74%). Of the participating children included in the analysis (91/210, 43.3%), the majority were male (45/85, 53% of the children from whom demographics were collected) with a mean age of 22 (SD 13) months, and 46% (39/85) of children reported to be aged between 1 and 2 years.

### Comparison of Nutrient Intake Between Recording Periods

Of the 119 participating mothers, 111 (93.3%) were included in all the 3 recording period comparisons, with the remainder 8 (6.7%) included in only 1 comparison between recording periods. Of the 91 participating children, 78 (86%) were included in all the 3 comparisons between recording periods, while 13 (14%) participants included in the analysis comparing 1 recording period. For each of the 3 comparisons, the number of participants included were (1) VISIDA period 1 versus 24-hour recalls: 117/119, 98.3% mothers and 86/91, 95% children; (2) VISIDA period 2 versus 24-hour recalls: 112/119, 94.1% mothers and 81/91, 89% children; and (3) VISIDA period 1 versus VISIDA period 2: 112/119, 94.1% mothers and 80/91, 88% children.

Tables 1 and 2 present the descriptive data and effect sizes for the pairwise comparison between the 3 recording periods for mothers and children, respectively. The linear mixed models that were fitted to the data satisfied the assumptions of normality and constant variance of residuals after appropriate weighting functions were applied to adjust for the nonconstant variability evident in all measures.

The effect sizes were differences in model-weighted marginal means for each pair of conditions. The differences in weighted means may differ somewhat from those calculated based on the raw data means in the tables. This variation reflects that in the calculation of the weighted marginal means, higher values were less important due to their higher inherent variability than the lower values that had lower variability.

Of the 20 nutrients analyzed for the mothers, statistically significant differences between the methods were found for 16 (80%) nutrients when both VISIDA recording periods were compared to 24-hour recalls (Table 1). For the children, intakes for 6 (32%) out of the 19 nutrients showed statistically significant differences between the VISIDA recording periods and 24-hour recalls (Table 2). In general, the mean intakes reported using the 24-hour recall were higher compared to either of the 2 VISIDA recording periods. When intakes estimated from both the VISIDA recording periods were compared, there were no statistically significant differences observed for either mothers or children.

**Table 1:** Summary statistics for the mothers and effect sizes as the difference between the weighted means for pairs of recording periods and 95% CIs.

Nutrient	Daily nutrient intake of mothers for each recording period			Effect size as difference of model-weighted marginal means			
	VISIDA^a^ period 1, mean (SD)—unadjusted	Interviewer-administered 24-h recall, mean (SD)—unadjusted	VISIDA period 2, mean (SD)—unadjusted	*P* value	VISIDA period 1 minus 24-hr recall, mean (95% CI)	VISIDA period 2 minus 24-hr recall, mean (95% CI)	VISIDA period 1 minus VISIDA period 2, mean (95% CI)
Energy (kcal)	1406 (643)	1712 (759)	1424 (664)	<.001	−296 (−410 to −181)	−274 (−390 to −158)	−22 (−131 to 87)
Protein (g)	60.3 (33.6)	66.9 (33.5)	63.7 (39.2)	.04	−6.6 (−11.7 to −1.4)	−4.7 (−10.0 to 0.6)	−1.9 (−6.8 to 3.1)
Fat (g)	47.9 (35.5)	54.9 (40.8)	47.1 (36.9)	.24	−3.9 (−9.3 to 1.6)	−4.4 (−9.8 to 1.0)	0.5 (−4.5 to 5.6)
Carbohydrates (g)	181.5 (84.1)	235.2 (103.5)	185.6 (84.4)	<.001	−46.4 (−60.3 to −32.4)	−42.6 (−56.8 to −28.4)	−3.8 (−16.8 to 9.2)
Dietary fiber (g)	9.3 (5.9)	10.9 (6.7)	9.3 (5.5)	.003	−1.5 (−2.4 to −0.6)	−1.3 (−2.2 to −0.4)	−0.2 (−1.0 to 0.7)
Alcohol (g)	0.2 (2.5)	1.5 (7.6)	0.6 (5.9)	.01^b^	−1.34 (−2.24 to −0.45)	−0.90 (−1.80 to 0.01)	−0.45 (−1.35 to 0.46)
Vitamin A RE^c^ (µg)	425.5 (412.3)	459.1 (375.2)	457.3 (676.4)	.009	−68.7 (−118.6 to −18.8)	−5.8 (−55.8 to 44.3)	−62.9 (−110.6 to 15.2)
Thiamine (mg)	0.8 (0.6)	0.8 (0.5)	0.8 (0.6)	.68	−0.03 (−0.11 to 0.06)	−0.04 (−0.12 to 0.05)	0.01 (−0.07 to 0.09)
Riboflavin (mg)	0.8 (0.5)	0.9 (0.5)	0.9 (0.5)	.03	−0.10 (−0.17 to −0.02)	−0.06 (−0.14 to 0.01)	−0.03 (−0.10 to 0.04)
Niacin (mg)	13.0 (8.1)	16.6 (12.6)	14.2 (11.8)	<.001	−3.4 (−4.7 to −2.1)	−2.7 (−4.1 to −1.4)	−0.7 (−1.8 to 0.4)
Vitamin B6 (mg)	1.3 (1.2)	1.9 (2.4)	1.5 (1.7)	.004	−0.21 (−0.34 to −0.09)	−0.16 (−0.29 to −0.03)	−0.06 (−0.15 to 0.04)
Vitamin B12 (µg)	4.4 (3.9)	5.6 (8.8)	4.9 (4.8)	<.001	−1.6 (−2.4 to −0.9)	−0.9 (−1.7 to −0.1)	−0.8 (−1.4 to −0.1)
Vitamin C (mg)	50.0 (63.3)	60.2 (69.0)	49.8 (51.5)	.04^b^	−10.6 (−20.2 to −1.1)	−10.8 (−20.4 to −1.1)	0.1 (−9.5 to 9.8)
DFE^d^ (µg)	336.0 (162.4)	409.0 (198.8)	340.6 (169.0)	<.001	−66.9 (−93.0 to −40.7)	−61.6 (−88.2 to −35.1)	−5.2 (−29.8 to 19.3)
Calcium (mg)	487.4 (531.1)	623.6 (595.6)	507.9 (434.0)	.004^b^	−136.6 (−222.5 to −50.6)	−116.6 (−203.7 to −29.5)	−20.0 (−106.8 to 66.9)
Phosphorus (mg)	782.9 (433.2)	855.0 (437.1)	796.1 (437.5)	.06	−71.0 (−134.8 to −7.3)	−64.5 (−129.5 to 0.5)	−6.5 (−69.0 to 55.9)
Sodium (mg)	3492.2 (2770.0)	3854.1 (2766.8)	3549.5 (2715.7)	.01	−457.6 (−793.1 to −122.0)	−451.9 (−789.9 to −114.0)	−5.7 (−314.5 to 303.2)
Potassium (mg)	1559.8 (872.4)	1722.5 (879.7)	1622.0 (904.1)	.01	−183.1 (−306.1 to −60.0)	−135.8 (−261.8 to −9.7)	−47.3 (−168.5 to 73.9)
Iron (mg)	11.0 (7.4)	14.3 (11.1)	12.2 (9.5)	<.001	−4.0 (−5.5 to −2.6)	−2.9 (−4.4 to −1.4)	−1.1 (−2.3 to 0.2)
Zinc (mg)	6.1 (3.3)	6.7 (3.5)	6.2 (3.5)	.12	−0.47 (−0.97 to 0.02)	−0.44 (−0.95 to 0.06)	−0.03 (−0.51 to 0.45)

^a^VISIDA: Voice-Image Solution for Individual Dietary Assessment.

^b^Estimates from a model where no weighting was used.

^c^RE: vitamin A retinol equivalents.

^d^DFE: dietary folate equivalents.

**Table 2:** Summary statistics for the children and effect sizes as the difference between the weighted means for pairs of recording periods and 95% CIs.

Nutrient	Child daily nutrient intake for each recording period				Effect size as difference of model weighted marginal means			
	VISIDA^a^ period 1, mean (SD)—unadjusted	Interviewer-administered 24-h recall, mean (SD)—unadjusted	VISIDA period 2, mean (SD)—unadjusted	*P* value		VISIDA period 1 minus 24-hr recall, mean (95% CI)	VISIDA period 2 minus 24-hr recall, mean (95% CI)	VISIDA period 1 minus VISIDA period 2, mean (95% CI)
Energy (kcal)	617 (442)	793 (524)	646 (447)	<.001		−158 (−227 to −89)	−127 (−198 to −57)	−31 (−98 to 37)
Protein (g)	26.3 (22.0)	25.4 (16.6)	26.7 (20.9)	.77		−1.0 (−3.7 to 1.7)	−0.4 (−3.1 to 2.4)	−0.6 (−3.4 to 2.1)
Fat (g)	21.8 (23.2)	27.0 (25.0)	22.1 (21.5)	.006		−3.1 (−5.1 to −1.1)	−2.8 (−4.8 to −0.8)	−0.3 (−2.0 to 1.4)
Carbohydrates (g)	78.7 (58.0)	111.1 (70.6)	83.8 (58.1)	<.001		−28.4 (−37.6 to −19.3)	−22.0 (−31.4 to −12.6)	−6.4 (−15.3 to 2.5)
Dietary fiber (g)	3.6 (3.1)	3.8 (3.7)	4.0 (3.6)	.55		−0.2 (−0.7 to 0.3)	0.1 (−0.4 to 0.6)	−0.3 (−0.8 to 0.2)
Vitamin A RE^b^ (µg)	190.2 (292.0)	194.0 (222.4)	213.4 (387.0)	.47		2.3 (−12.8 to 17.3)	12.7 (−7.1 to 32.5)	−10.5 (−30.3 to 9.4)
Thiamine (mg)	0.3 (0.4)	0.4 (0.3)	0.3 (0.3)	.02		−0.06 (−0.10 to −0.02)	−0.03 (−0.07 to 0.01)	−0.03 (−0.06 to 0.01)
Riboflavin (mg)	0.4 (0.4)	0.6 (0.7)	0.5 (0.5)	.03		−0.06 (−0.10 to −0.02)	−0.04 (−0.08 to 0.01)	−0.02 (−0.07 to 0.02)
Niacin (mg)	5.2 (4.2)	5.5 (4.7)	5.3 (4.2)	.56		−0.25 (−0.75 to 0.25)	−0.23 (−0.73 to 0.28)	−0.02 (−0.52 to 0.48)
Vitamin B6 (mg)	0.5 (0.4)	0.5 (0.7)	0.5 (0.4)	.36		−0.05 (−0.11 to 0.02)	−0.02 (−0.08 to 0.04)	−0.03 (−0.09 to 0.04)
Vitamin B12 (µg)	1.8 (2.2)	1.7 (2.1)	2.0 (2.2)	.40		0.06 (−0.14 to 0.26)	0.14 (−0.07 to 0.35)	−0.09 (−0.31 to 0.13)
Vitamin C (mg)	18.7 (24.0)	20.9 (37.1)	22.7 (30.4)	.98		0.11 (−1.51 to 1.73)	0.17 (−1.64 to 1.99)	−0.06 (−1.77 to 1.65)
DFE^c^ (µg)	143.5 (108.2)	175.4 (153.0)	154.2 (113.1)	.06		−21.8 (−40.2 to −3.4)	−15.4 (−34.2 to 3.4)	−6.5 (−25.0 to 12.0)
Calcium (mg)	217.5 (254.6)	273.6 (252.9)	230.9 (211.3)	.04		−23.7 (−41.9 to −5.4)	−13.6 (−32.7 to 5.5)	−10.1 (−26.8 to 6.6)
Phosphorus (mg)	340.9 (276.3)	363.6 (254.9)	348.5 (258.3)	.24		−29.2 (−63.8 to 5.5)	−20.5 (−55.9 to 14.8)	−8.6 (−43.8 to 26.5)
Sodium (mg)	1363.0 (1276.9)	1251.2 (1082.8)	1356.1 (1441.9)	.08		−68.7 (−162.3 to 25.0)	−100.0 (−189.8 to −10.1)	31.3 (−63.0 to 125.7)
Potassium (mg)	657.1 (550.6)	675.3 (515.0)	655.3 (517.2)	.37		−43.6 (−114.7 to 27.5)	−44.4 (−116.3 to 27.5)	0.8 (−70.9 to 72.6)
Iron (mg)	4.9 (4.7)	5.0 (4.5)	4.9 (4.3)	.15		−0.41 (−0.83 to 0.02)	−0.31 (−0.74 to 0.12)	−0.10 (−0.52 to 0.32)
Zinc (mg)	2.5 (1.9)	2.5 (1.8)	2.7 (2.0)	.77		−0.05 (−0.30 to 0.20)	0.05 (−0.21 to 0.30)	−0.09 (−0.35 to 0.17)

^a^VISIDA: Voice-Image Solution for Individual Dietary Assessment.

^b^RE: vitamin A retinol equivalents.

^c^DFE: dietary folate equivalents.

### Acceptability of the VISIDA Smartphone App for Collecting Dietary Intake Data

**Figure 2:**
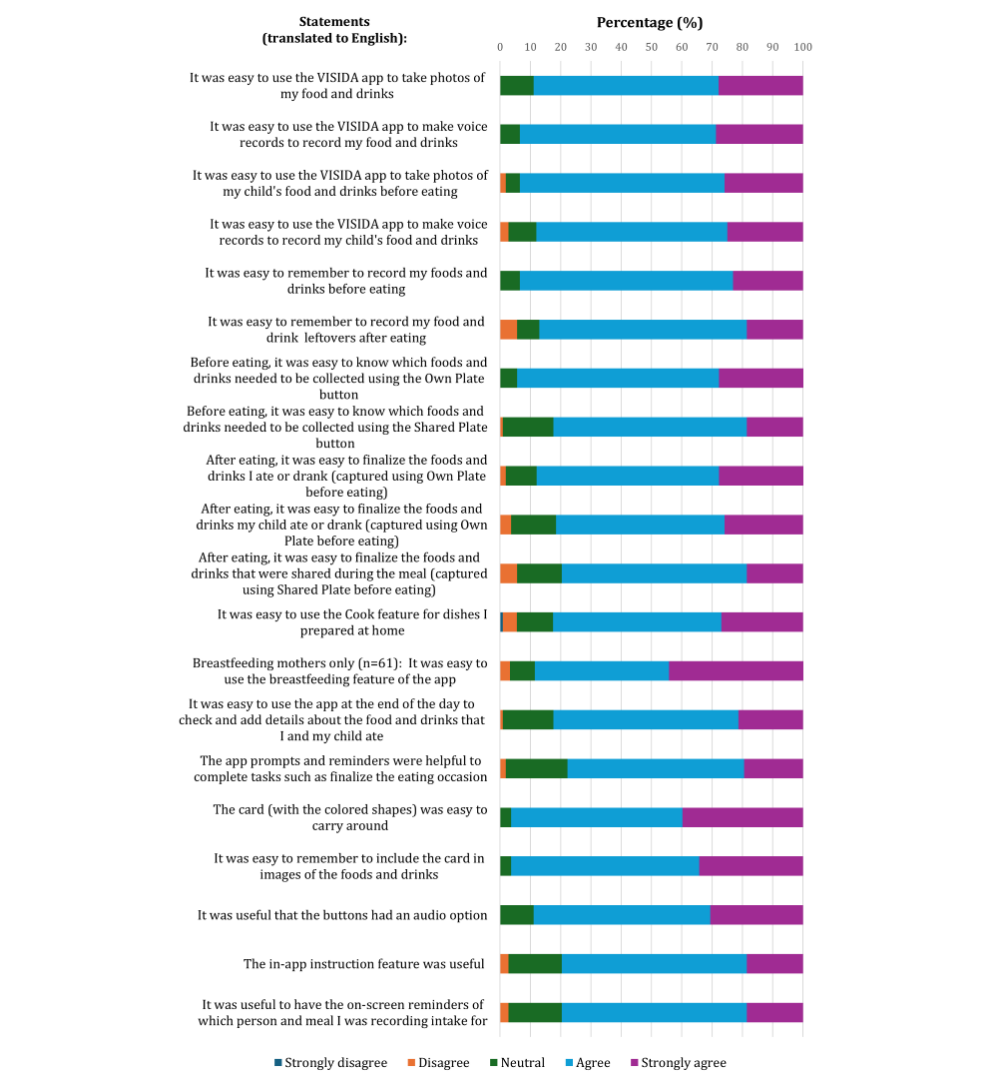
Acceptability of the Voice-Image Solution for Individual Dietary Assessment (VISIDA) smartphone app for collecting dietary intake data among the participating mothers (n=108).

The participating mothers completed the questionnaire on acceptability of the VISIDA IVFR app at the end of the study, with 108 responses captured (Figure 2). Overall, the participants reported that the app as “easy to use” (68/108, 63%), followed by “very easy to use” (23/108, 21.3%), “neutral” (9/108, 8.3%), “difficult to use” (7/108, 6.5%), and “very difficult to use” (1/108, 0.9%). Most participants “agreed,” followed by “strongly agreed” for all statements. The mean score for all statements was 4.1 (SD 0.14; out of 5), with the mean scores for individual statements ranging from 3.9 (SD 0.7; out of 5) for “After eating, it was easy to finalize the foods and drinks that were shared during the meal (captured using Shared Plate before eating)” and “The app prompts and reminders were helpful to complete tasks like finalize eating occasion” to 4.4 (SD 0.6; out of 5) for “The card (with the colored shapes) was easy to carry around.” All participants indicated that they would be willing to use the app again, with 81.5% (88/108) of the participants reporting that they would be willing to use the app for “1 month or more,” followed by “1 week” (13/108, 12%), “1 day” and “2 days” (3/108, 2.8%, each) and “3 days” (1/108, 0.9%).

## Discussion

### Principal Findings

We evaluated the relative validity, test-retest reliability, and acceptability of the novel VISIDA system for individual dietary assessment in mothers and children (aged ≤5 years) in rural, semirural, and urban locations in Siem Reap province, Cambodia. The findings demonstrated that most nutrient intakes estimated by the VISIDA system (test method) were significantly lower compared to intakes estimated using the 24-hour recalls (reference method) for the participating mothers and their child. However, nutrient intakes estimated by the VISIDA system in the 2 recording periods that used this method were similar. The VISIDA IVFR app was consistently rated highly for acceptability by the participating mothers.

### Comparison to Previous Work

This study is the first to validate an image-based method to assess dietary intakes of mothers and their children in Cambodia. To the best of our knowledge, only 2 studies have validated dietary assessment methods in this context: a food frequency questionnaire in school-aged children [45] and 2 proxy recall approaches for the estimation of diet diversity in women of reproductive age [46]. Horiuchi et al [45] reported that nutrient intakes estimated using the food frequency questionnaire were lower compared to 24-hour recalls. In contrast, Hanley-Cook et al [46] found that both the list-based and open recall approaches were similar in estimating the minimum diet diversity. However, both proxy recall approaches showed lower correlations compared to weighed food records [46]. Nutrient intakes were higher when estimated by the 24**-**hour recall method compared to the VISIDA image-based system. This finding aligns with a recent meta-analysis of image-based dietary assessment methods [11], which reported a tendency to report lower-energy intakes using this method in comparison to 24-hour recalls (mean difference −91.6 kcal) and weighed food records (mean difference −52.6 kcal).

In this study, greater differences in energy intakes were observed between the 2 VISIDA record periods and 24-hour recalls collection, with differences of approximately −300 kcal for mothers and −150 kcal for children. The difference in energy intake between the VISIDA and 24-hour recalls appears to be driven by the difference in the estimates of carbohydrate intake, which displayed the largest difference compared to the estimates of protein and fat intakes. In their systematic review, Ho et al [11] also found that the greatest difference between image-based and traditional 24-hour and weighed food record methods was for estimates of carbohydrate intake; however, this difference was not statistically significant and was smaller compared to our study.

While exploration of the sources of difference in the estimates of nutrient intake between methods is beyond the scope of this study, possible areas that may have influenced this finding can be proposed. Key differences between the VISIDA and 24-hour recall methods exist in the quantification of food consumed, which may have contributed to this difference between the methods. For the VISIDA method, the responsibility for quantification lies with the trained analyst supported by various tools, such as a reference image database, a measures database, and a virtual ruler, within the VISIDA web application. In comparison, for the 24-hour recall method, the participating mothers were responsible for the estimation of portions of food consumed by themselves and their child, recalling these with the assistance of images of common foods and eating and serving utensils. Portion size estimation in Asian cuisines can be challenging due to the foods consumed predominantly being amorphous, taking on the shape of the vessel in which they are served, and foods commonly eaten in shared servings [47]. Adding to this challenge, amorphous and nonamorphous foods are commonly consumed together in the same vessel [48]. Given that rice is a staple of the Cambodian diet [18], it is possible that the 2 approaches to quantifying rice portions consumed may be responsible for the differences in carbohydrate intake observed between the methods in our study.

Furthermore, in this study, the estimations of the amounts of food participants consumed from a vessel shared by more than one individual were quantified differently by each method. For the 24-hour recall method, the mothers were asked to quantify the amount consumed by themselves and their child for shared servings of food. In comparison, the VISIDA method estimated the quantity consumed from each shared food serving automatically by taking the total quantity of the shared dish consumed and dividing it by the number of people who ate the shared dish. It is possible that this difference in estimating individual portions consumed from shared dishes between methods may explain some of the variation observed in the estimations of nutrient intake. For cultures where eating from communal servings is common, apportioning amounts consumed by an individual remains a challenge [26,27]. Efforts to improve estimates of individual portion size from shared foods in the context of dietary assessment must be balanced with consideration of the cumulative burden placed on participants, which in turn may promote reactivity or changes to eating behaviors that are not reflective of typical intake to facilitate recording [49].

The repeat administration of the VISIDA method in this sample of Khmer women and children showed small, mostly nonstatistically significant differences in the estimates of nutrient intake made in weeks 1 and 4. Reliability is an important component to establish when evaluating a method’s validity, with the data on both aspects to be considered when determining the suitability of a dietary assessment method for use in a given setting [21]. Therefore, while it is important to establish the reliability of the VISIDA system, particularly in the context of the first application of the system in measuring individual-level dietary intakes, the reliability findings should be interpreted alongside the relative validity results where differences were present in the estimates of nutrient intakes made by the VISIDA system compared to estimates derived from the 24-hour recalls. Furthermore, it is important to highlight that the VISIDA system should be tested in other LLMICs, including with different population subgroups, and evaluated against objective measures to provide additional insights into the performance of this novel method.

One of the advantages of image-based food records over traditional written or text-based food records is that the participant burden associated with recording, such as weighing foods, is often reduced when images are collected to capture dietary intake data. The VISIDA system offers a novel component through the inclusion of the voice recording component, in addition to the collection of images. The aim of including the voice component was to facilitate the collection of additional intake data relatively quickly through speech and to reduce reliance on the literacy skills needed for using smartphone apps for text-based food record entry [50]. While image-based and speech-based methods can reduce participant burden associated with the collection of intake data, approaches to the processing of this data to extract the identity and quantity for each food to enable the estimation of nutrient intake are an important consideration. In addition, manual image processing can facilitate immediate application in research settings, while approaches offering automation require further development [51].

Despite substantial progress in the use of computer vision and machine learning in the automatic processing of image-based food records, challenges (in particular for quantification) still remain when used in free-living situations due to the variability and complexity of the meals consumed and the environment in which the images are collected [52]. While technologies to support the automated identification and quantification of foods contained in images continue to advance, there is still a need for verification by a human of the automated machine generated outputs of the analysis of food images used in dietary intake assessment, with this verification likely to be required for the foreseeable future [12]. Each dietary assessment method has strengths and weaknesses [53], and the VISIDA system is no different. In particular, the time and resource implications of data processing are considerable, which would limit the feasibility of using the current VISIDA system for large population surveys at this time. Therefore, use of the current VISIDA system may be more feasible for targeted data collection projects where detailed information on recipes prepared in the home is required or where intake data are required for smaller groups of individuals.

While most image and voice intake data processing within the VISIDA system was performed by trained analysts, some key tasks were semiautomated. For example, the VISIDA system’s voice recording was automatically transcribed and translated, with key terms then matched to the food items contained in the FCD and offered as suggestions to the analyst. Measuring the impact of the automated processing of the voice recording component of intake data was not part of this study. However, enhancing understanding of the benefits and drawbacks that the different levels of processing automation provide, in terms of accuracy, efficiency, and resource use, in the context of image-based and speech-based dietary intake assessments, warrants further investigation.

Among the participating mothers, high acceptability was reported for use of the VISIDA IVFR app to collect eating occasions for themselves and their participating child, along with recipe data for meals prepared at home. Evaluating participant acceptability and usability is an important facet of technology-assisted self-report dietary assessment methods used for research or surveillance [8]. When usability and acceptability have been assessed for image-based food records or intake data captured via speech recordings as independent dietary assessment methods, most participants consistently rate these tools positively [12,16]. Previous dietary assessment studies in Cambodia have used interview-assisted methods of data collection, with the majority using “pen and paper” tools for documentation [18]. Findings from this study provide useful insights into the potential of using a self-administered tool to collect intake data in Cambodia. In addition, these findings align with similar findings from our earlier work relating to image-based food records being viewed positively by participants [24,30], including a more recent study in which the VISIDA IVFR app was used by Tanzanian nutritionists [54].

### Strengths and Limitations

This study has several strengths and limitations that should be considered when interpreting the findings. First, the study design, including the implementation of the test method (VISIDA) before the reference method (24-hour recall), the repeat administration of the VISIDA method, and the multiple days of intake data for each method, is a strength. As a reference method in studies evaluating image-based food records, 24-hour recalls are commonly used [11]. However, as a self-report method, it is not considered unbiased [21], and our findings should be interpreted in this context. Second, the inclusion of a test recording day to allow the research team to support participants in using the VISIDA IVFR app for the first time and collect intake data in their home, aimed to aid users and optimize the quality of the data collected. Despite the detailed training and addition of the test recording day to support participating mothers in using the IVFR app, it is likely that not all intake data were captured during data collection. A review of dietary intake data was included following each day of the data collection to optimize the completeness of the data collected. Third, the COVID-19 pandemic at the start of 2020 resulted in the early cessation of data collection for 19 households (consisting of mother and child), which impacted the final number of participants included in this analysis. While it is unclear whether a larger sample would produce different results, in the context of dietary assessment validation studies, our study is one of the largest image-based food record validation studies, with the sample size of previous studies ranging from 10 to 75 participants [11]. We also recruited participants from 3 different locations within Siem Reap province, resulting in a more diverse sample. However, participants in this study may not be representative of mothers and children aged ≤5 years in other provinces throughout Cambodia. Thus, inferences cannot be made about the potential performance of the VISIDA system when used with individuals, groups, and contexts beyond those examined in this study. Fourth, it is possible that because the questionnaire on the acceptability of the VISIDA IVFR app was administered by a research assistant, this may have influenced the responses received. However, given the setting, this was determined to be the most suitable approach.

### Conclusions

When evaluated in a sample of mothers and their children aged ≤5 years in rural, semirural, and urban locations in Siem Reap province, Cambodia, the VISIDA system was found to produce lower estimates of nutrient intakes when compared to the 24-hour recalls. However, on repeat administration of the VISIDA system, estimated nutrient intakes were similar. Participating mothers reported high acceptability for using the VISIDA IVFR smartphone app to collect dietary intake data.

## Appendix

Multimedia Appendix 1 Participant data collection sequence.

Multimedia Appendix 2 STROBE-nut (Strengthening the Reporting of Observational Studies in Epidemiology-Nutritional Epidemiology) checklist.

## Abbreviations

FCD: food composition database
IVFR: image-voice food record
LLMIC: low- and lower-middle-income country
NGO: nongovernmental organization
VISIDA: Voice-Image Solution for Individual Dietary Assessment

## Data Availability

The datasets generated or analyzed during this study may be made available from the corresponding author upon reasonable request.
