# Supplementary material for: An Image-Voice Dietary Assessment System for Estimating Individual Nutrient Intakes in Cambodian Women and Children: Relative Validity, Reliability, and Acceptability Study
Source: J Med Internet Res. 2025 Sep 17;27:e65939. doi: 10.2196/65939 (PMC12489410; doi:10.2196/65939)
Supplement: Multimedia Appendix 1 [file jmir_v27i1e65939_app1.pdf]

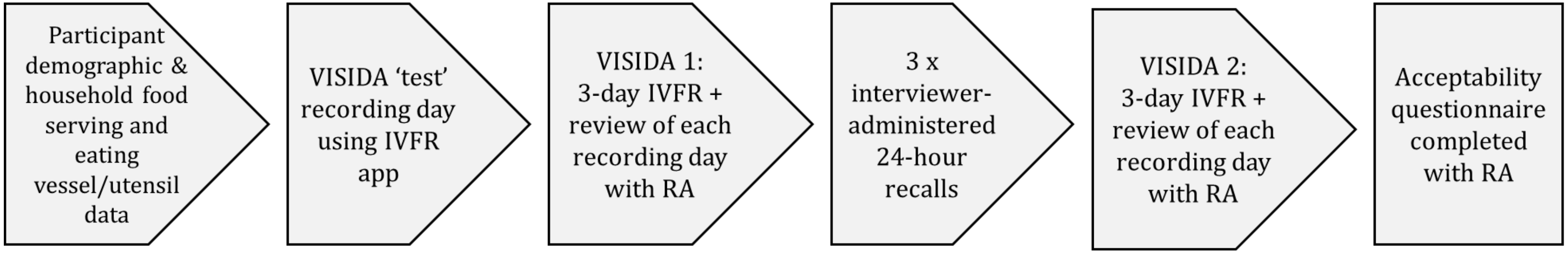

Abbreviations: IVFR, image-voice food record; RA, Research Assistant; VISIDA 1, first VISIDA recording period; VISIDA 2, second VISIDA recording period.

Multimedia Appendix 1. Participant data collection sequence. Data collection occurred over a period of approximately 4 weeks. At the start of data collection period, demographic and household inventory data on the food serving and eating vessels/utensils were collected. This was followed by the first recording period using the VISIDA IVFR app (VISIDA 1) where the mother collected dietary intake data for herself and her child over 3 days in Week 1. After each IVFR recording day, a research assistant visited the household to review the data collected. In Weeks 2-3, the research assistants completed three interviewer-administered 24-hour recalls for each participant. In Week 4, the VISIDA IVFR app was used again to collect intake data (VISIDA 2), following the same protocol as the first VISIDA recording period. After the second VISIDA recording period, the participating mother completed a brief questionnaire on the experience of using the VISIDA IVFR app to collect intake data which was administered by a research assistant.
